# Detecting O_2_ binding sites in protein cavities

**DOI:** 10.1038/srep20534

**Published:** 2016-02-02

**Authors:** Ryo Kitahara, Yuichi Yoshimura, Mengjun Xue, Tomoshi Kameda, Frans A. A. Mulder

**Affiliations:** 1 College of Pharmaceutical Sciences, Ritsumeikan University, Nojihigashi 1-1-1, Kusatsu 525-8577, Japan; 2 Department of Chemistry and Interdisciplinary Nanoscience Center (iNANO), University of Aarhus, Gustav Wieds Vej 14, DK-8000 Aarhus C, Denmark; 3 Biotechnology Research Institute for Drug Discovery, Advanced Industrial Science and Technology (AIST), 2-43 Aomi, Koto, Tokyo 135-0064, Japan

## Abstract

Internal cavities are important elements in protein structure, dynamics, stability and function. Here we use NMR spectroscopy to investigate the binding of molecular oxygen (O_2_) to cavities in a well-studied model for ligand binding, the L99A mutant of T4 lysozyme. On increasing the O_2_ concentration to 8.9 mM, changes in ^1^H, ^15^N, and ^13^C chemical shifts and signal broadening were observed specifically for backbone amide and side chain methyl groups located around the two hydrophobic cavities of the protein. O_2_-induced longitudinal relaxation enhancements for amide and methyl protons could be adequately accounted for by paramagnetic dipolar relaxation. These data provide the first experimental demonstration that O_2_ binds specifically to the hydrophobic, and not the hydrophilic cavities, in a protein. Molecular dynamics simulations visualized the rotational and translational motions of O_2_ in the cavities, as well as the binding and egress of O_2_, suggesting that the channel consisting of helices D, E, G, H, and J could be the potential gateway for ligand binding to the protein. Due to strong paramagnetic relaxation effects, O_2_ gas-pressure NMR measurements can detect hydrophobic cavities when populated to as little as 1%, and thereby provide a general and highly sensitive method for detecting oxygen binding in proteins.

Internal cavities in proteins are important structural elements that may produce functional motions^1^, such as drug and ligand binding^2^ and conformational transitions into high-energy states^3^,^4^,^5^,^6^,^7^. To explore their locations and dynamic aspects, specific binding of noble gases, particularly xenon, into protein cavities has been studied by X-ray crystallography^8^,^9^,^10^. In addition, small organic compounds and paramagnetic agents as well as noble gases have been used as probes in various nuclear magnetic resonance (NMR) studies^11^,^12^,^13^,^14^,^15^. The fact that small organic compounds and noble gases can associate with internal cavities indicates that proteins are sufficiently dynamic to enable the access of small molecules and that cavities may function as gateways for them.

Penetration of dissolved oxygen (O_2_) into proteins was originally investigated by quenching of fluorescence^16^,^17^. The paramagnetic effects of O_2_, such as paramagnetic shifts and paramagnetic relaxation enhancements (PREs), have been used to study protein solvent exposure and topology by NMR spectroscopy^18^,^19^,^20^,^21^. Although many crystal structures of heme-proteins with O_2_ ligands and their migration processes inside the proteins have been investigated by X-ray crystallography^22^,^23^ and molecular dynamics (MD) simulation^24^,^25^, to the best of our knowledge, association of O_2_ with internal cavities of proteins in solution has been investigated by NMR spectroscopy only for ribonuclease A^12^,^26^, deoxymyoglobin^13^, and the B domain of protein A^20^. In particular, Teng and Bryant investigated O_2_-induced PREs for backbone and side chain protons of ribonuclease and showed that structural fluctuations in the protein provide access to the protein interior for O_2_. However, the O_2_-induced PREs were not simply correlated with the depth of a buried proton or hydrophobicity indices. Rather, large PREs were correlated with the distance to the closest hydrophobic cavity^12^,^20^. These studies suggest that O_2_ represents a useful paramagnetic NMR probe to explore the surface crevices and cavities of proteins, which have the potential for ligand binding.

By using gas-pressure NMR and MD simulation, we investigate O_2_ accessibility to the protein interior for the cavity-enlarged L99A mutant of T4 lysozyme, which has two hydrophilic cavities (cavity 1: 50 Å^3^, cavity 2: 25 Å^3^) and two hydrophobic cavities (cavity 3: 25 Å^3^, cavity 4: 150 Å^3^). Cavity 4 was enlarged from 39 Å^3^ to 150 Å^3^ by the Leu → Ala mutation at position 99^27^. The L99A mutant has been used as a model system for understanding protein dynamics in the ligand binding process. X-ray crystallography found that three xenon atoms are present in cavity 4 under 8 bar of xenon pressure, and cavity 4 has been shown to allow the binding of benzene and substituted benzenes^10^,^28^,^29^. Although X-ray crystallography suggested that the enlarged cavity in L99A is sterically inaccessible to incoming ligands, NMR spin relaxation studies showed the presence of conformational fluctuations around the hydrophobic cavities and the rapid exchange of benzene and indole with the protein interior^30^,^31^,^32^,^33^,^34^. Our objective here is to understand the selectivity of O_2_ to hydrophilic and hydrophobic cavities and the coupling between protein conformational fluctuation and accessibility of O_2_ to internal cavities of the protein.

## Results and Discussion

### Reversible association of oxygen

We used on-line gas pressure NMR spectroscopy up to 7 bar absolute pressure to demonstrate gas binding into cavities of the cavity-enlarged L99A mutant of the T4 lysozyme. Time-dependent changes in ^1^H NMR spectra of the protein were observed, when the concentration of molecular oxygen (O_2_) decreases from 1.8 mM (corresponding to 1.4 bar absolute pressure) to 0.27 mM (corresponding to atmospheric pressure; 0.2 bar O_2_ partial pressure) at 298 K. A well-separated peak stemming from L121 Hδ_1_ changed its frequency (about 0.05 ppm) during 18.7 hours after pressure decreased, and the chemical shift change during the final hour was 0.0009 ppm, which is at the level of indiscernible changes in chemical shifts (^1^H: ±0.001 ppm). Therefore, we regarded that 18.7 hours is sufficient to reach a new equilibrium of gas dissolution in the NMR tube (see Supplementary Fig. S1 online). All NMR measurements were started more than 18.7 hours after gas pressure was changed. In the present pressure range, spectral changes were perfectly reversible.

### Oxygen-induced spectral changes

**Figure 1:**
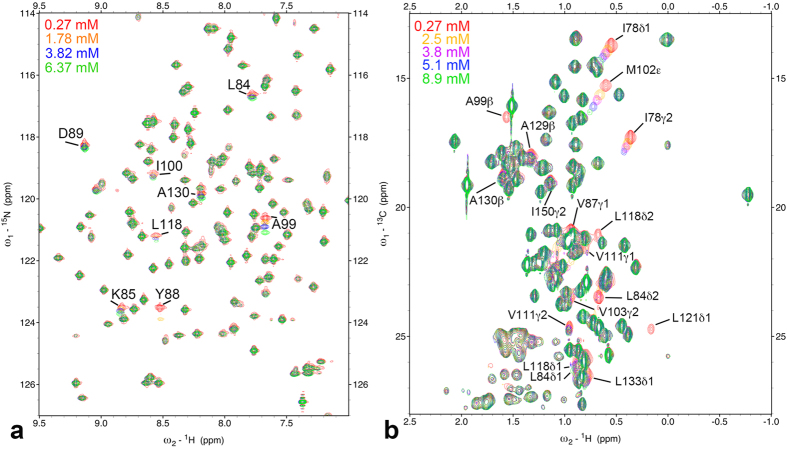
(**a**) ^1^H/^15^N refocused-HSQC spectra of ^15^N labeled L99A of T4 lysozyme at 298 K at different oxygen concentrations from 0.27 mM to 6.4 mM. Amide groups showing significant changes in ^15^N chemical shift are indicated. (**b**) ^1^H/^13^C constant time HSQC spectra of ^13^C/^15^N labeled L99A of T4 lysozyme at different oxygen concentrations from 0.27 mM to 8.9 mM. Positive and negative crosspeaks are presented by same color. Methyl groups showing significant changes in ^1^H/^13^C chemical shift and a loss of crosspeak intensities are indicated.

Figure 1a shows ^1^H/^15^N refocused heteronuclear single-quantum coherence (HSQC) spectra of ^15^N-labeled L99A at O_2_ concentrations from 0.27 mM to 6.4 mM. O_2_-induced chemical shift changes were observed for cross-peaks of L84, K85, Y88, D89, A99, I100, L118, and A130. At 6.4 mM of O_2_, their O_2_-induced ^15^N chemical shifts are about 0.1–1.0 ppm. In addition, cross-peaks for Y88, I100, and L118 became weaker or disappeared with increasing O_2_ concentration. In contrast, N_2_ and Ar gas did not induce perceptible changes in chemical shifts and cross-peak intensities in the same ranges of gas concentrations (N_2_ ~3.3 mM, Ar ~7 mM; see Supplementary Fig. S2 online).

Figure 1b shows the region for methyl group signals in ^1^H/^13^C constant time (CT) HSQC spectra of ^15^N/^13^C-labeled L99A. O_2_-induced chemical shift changes and/or loss of signal intensities are significant for the methyl groups of I78δ_1_, I78γ_2_, L84δ_1_, L84δ_2_, V87γ_2_, A99β, M102ε, V103γ_2_, V111γ_1_, V111γ_2_, L118δ_1_, L118δ_2_, L121δ_1_, A129β, A130β, L133δ_1_, and I150γ_2_. In contrast, chemical shift changes were not observed when Ar was increased to 5 bar (~7 mM) or N_2_ was increased to 7 bar (~4.6 mM, see Supplementary Fig. S3 online).

**Figure 2:**
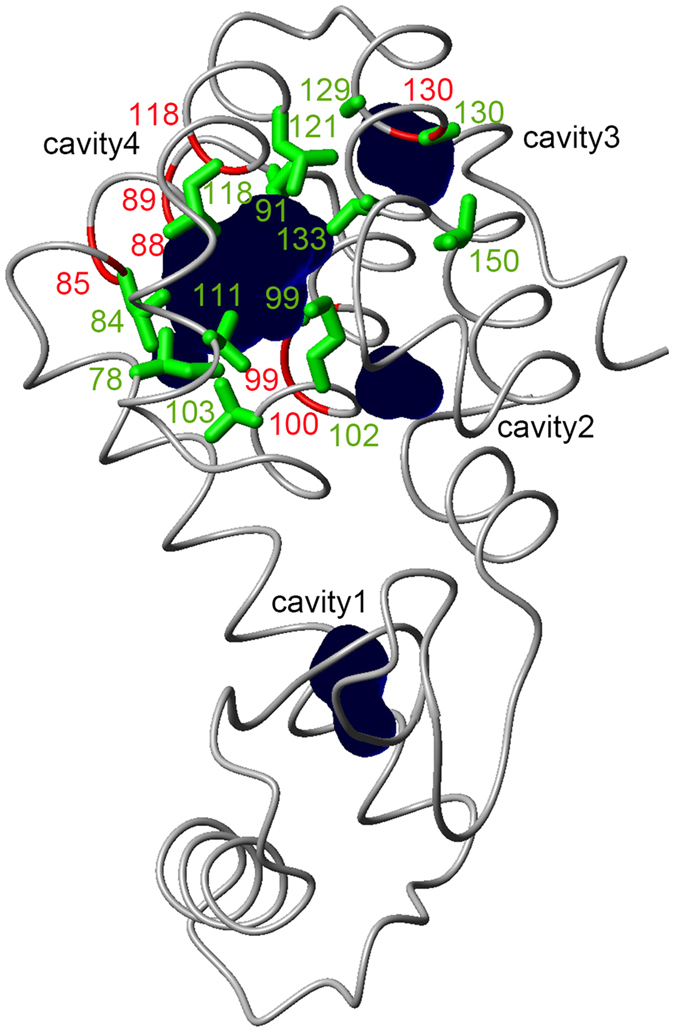
Three-dimensional structure representations of T4 lysozyme L99A, highlighting the locations of amide (red) and methyl (green) groups showing oxygen-induced chemical shift changes or a loss of crosspeak intensity. Cavities calculated by the program MOLMOL^62^ are depicted by dark blue spheres.

Figure 2 shows the mapping of backbone amide groups and methyl groups showing O_2_-induced changes in chemical shifts and/or cross-peak intensities. O_2_-induced changes are specific around the two hydrophobic cavities 3 and 4 in the C-terminal domain of the protein. These results suggest that O_2_ associated with cavities 3 and 4, and the O_2_-induced changes resulted from the paramagnetic property of O_2_ and/or changes in the structure and conformational equilibrium of the protein. Details are discussed further in the sections *The paramagnetic effect leads to line broadening* and *Origin of O*_*2*_*-induced chemical shift changes*.

**Figure 3:**
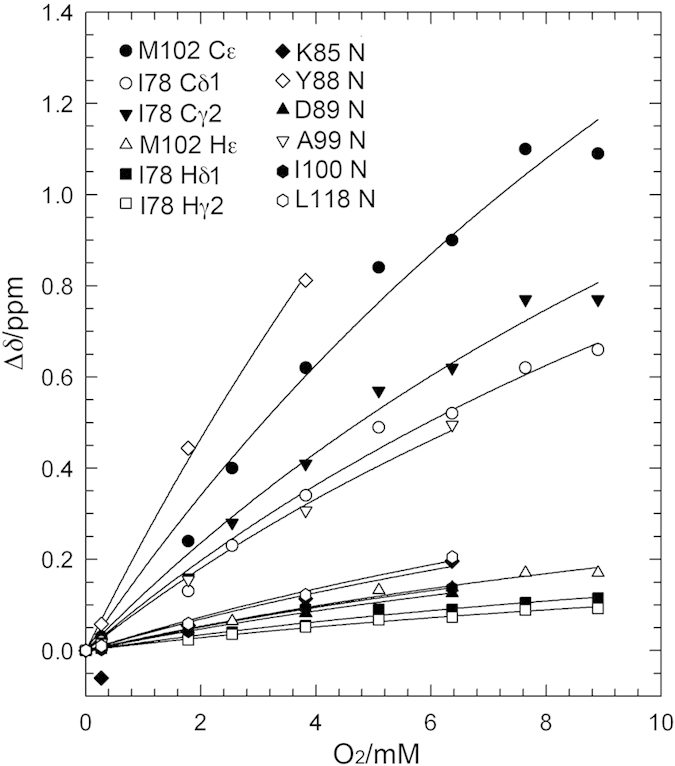
Chemical shift changes of methyl carbons, methyl protons, and amide nitrogens around the enlarged cavity as a function of O_2_ concentration.

Figure 3 shows the O_2_-induced chemical shift changes observed for amide nitrogens, methyl carbons, and methyl proton nuclei of the residues around the enlarged hydrophobic cavity (cavity 4). The O_2_ association constant can be estimated from the concentration dependence of peak positions, assuming exchange between O_2_-bound and free states. Global fitting to all chemical shift changes was performed, using the following equation (1),


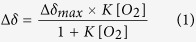


where [O_2_] is the molar concentration of dissolved O_2_, Δ*δ*_max_ is the residue-specific saturation value of the O_2_-induced shift, and *K* is the association constant, which is a global variable in the model fitting to data for all residues simultaneously. The deviations between actual measurements and predicted values from the fit seem to stem from inaccuracies in chemical shift determination. The dissociation constant *K*_d_ is the reciprocal of *K. K* and *K*_d_ were 48 ± 7 M^−1^ and 21 ± 3 mM, respectively. Accordingly, 1.3% of T4 lysozyme L99A was in the O_2_-bound state at atmospheric pressure (i.e., O_2_ concentration 0.27 mM). Δ*δ*_max_ for each site is summarized in Table 1. For the other hydrophobic cavity (cavity 3), we did not have sufficient data to estimate *K*.

### Relaxation enhancement is due to PRE

**Figure 4:**
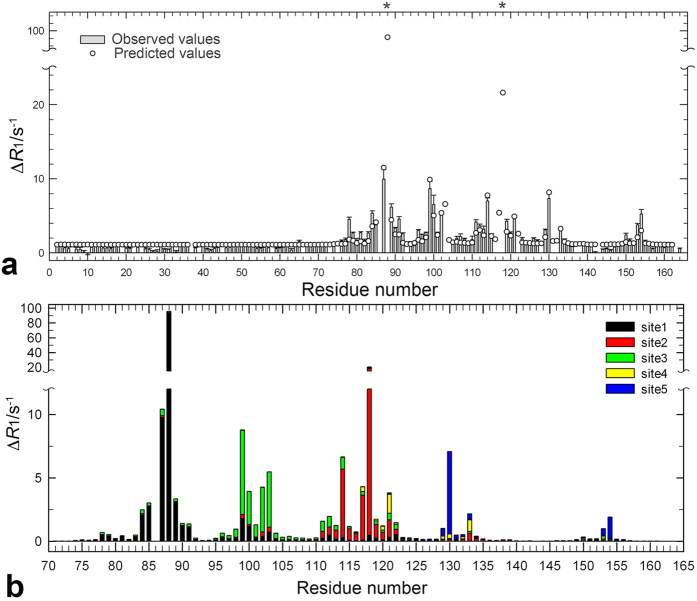
(**a**) Observed and predicted O_2_-induced ^1^H longitudinal relaxation enhancements for amide protons against residue number. Difference of longitudinal relaxation rates, Δ*R*_1_, for amide protons between 6.4 mM (O_2_ 5 bar) and 0 mM (Ar 2 bar) O_2_ concentrations. *R*_1_ values at each condition are shown in Supplementary Fig. S4 online. Severe line-broadening prohibited quantitative evaluation of Δ*R*_1_ for residues 88 and 118 (asterisks). The crystal structure of L99A at 8 atm of xenon pressure possesses three xenon molecules in cavity 4. We added two xenon molecules in cavity 3 and energy minimized. Δ*R*_1_ were estimated from 1/*r*^6^ weighted distance dependence from each xenon site, using the equation (2). (**b**) Contributions of each O_2_-binding site to the predicted ^1^H longitudinal relaxation enhancements for amide protons. Δ*R*_1_ from sites 1–5 were estimated by the following equation: (Δ*R*_1(predict)_-*f*) × (*a* or *b* or *c* or *d* or *e* × 10^5^ (1/*r*_1–5_)^6^)/(*a* × 10^5^ (1/*r*_*1*_)^6^ + *b* × 10^5^ (1/*r*_*2*_)^6^ + *c* × 10^5^ (1/*r*_*3*_)^6^ + *d* × 10^5^ (1*/r*_*4*_)^6^ + *e* × 10^5^ (1*/r*_*5*_)^6^), where *r*_*1–5*_ are distances to each xenon site. The parameters *a, b, c, d* and *e* were obtained to be 1.3, 1.1, 1.5, 0.11, 0.10, respectively, by the fitting.

**Figure 5:**
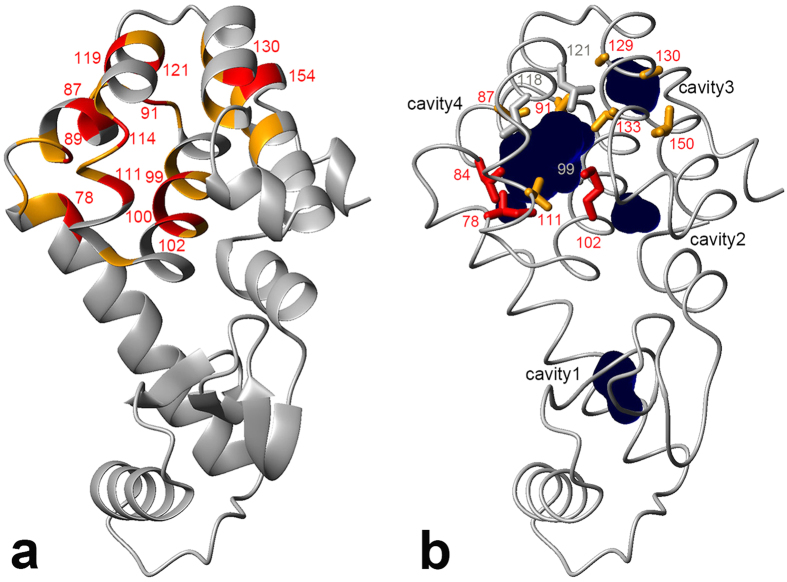
(**a**) Mapping of amide groups showing ^1^H longitudinal relaxation enhancements (Δ*R*_1_ ≥ 4 s^−1^, red; 2 s^−1^ ≤ Δ*R*_1_ < 4 s^−1^, orange). Data were obtained at 6.4 mM of O_2_ concentration. Amide groups showing large relaxation enhancements (Δ*R*_1_ ≥ 4 s^−1^) are labeled with residue number. (**b**) Mapping of methyl groups showing ^1^H longitudinal relaxation enhancements (Δ*R*_1_ ≥ 10 s^−1^, red; 4 s^−1^ ≤ Δ*R*_1_ < 10 s^−1^, orange). Data were obtained at 3.8 mM of O_2_ concentration. Methyl groups showing severe line-broadening are depicted by gray sticks. The picture was prepared using MOLMOL^62^.

**Figure 6:**
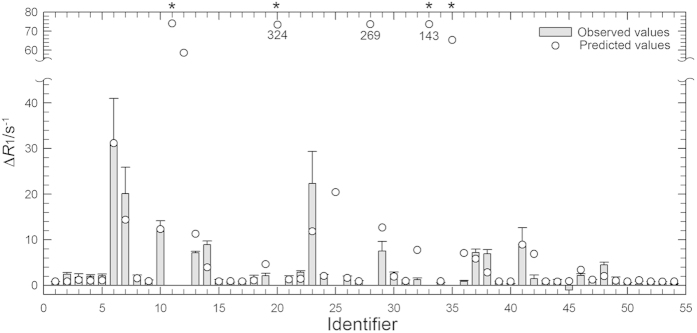
Observed and predicted O_2_-induced ^1^H longitudinal relaxation enhancements for methyl protons. Difference of longitudinal relaxation rates, Δ*R*_1_, for methyl protons between 3.8 mM (O_2_ 3 bar) and 0 mM (N_2_ 3 bar) O_2_ concentrations. *R*_1_ values at each condition are listed in Supplementary Table S1. Severe line-broadening prohibited quantitative evaluation of Δ*R*_1_ for L84δ_2_, A99β, L118 δ_2_, and L121 δ_1_ (asterisks). The crystal structure of L99A at 8 atm of xenon pressure possesses three xenon molecules in cavity 4. We added two xenon molecules in cavity 3 and energy minimized. Δ*R*_1_ were estimated from 1/*r*^6^ weighted distance dependence from each xenon site, using equation (2). Identifiers 1–54 have the following assignments: 1:V71γ_2_, 2:A73β, 3:A74β, 4:V75γ_1_, 5:V75γ_2_, 6:I78γ_2_, 7:I78δ_1_, 8:L79δ_2_, 9:A82β, 10:L84δ_1_, 11:L84δ_2_, 12:V87γ_1_, 13:V87γ_2_, 14:L91δ_2_, 15:A93β, 16:V94γ_1_, 17:V94γ_2_, 18:A97β, 19:A98β, 20:A99β, 21: I100γ_2_, 22:I100δ_1_, 23:M102ε, 24:V103γ_1_, 25:V103γ_2_, 26:M106ε, 27:T109γ_2_, 28:V111γ_1_, 29:V111γ_2_, 30:A112β, 31:T115γ_2_, 32:L118δ_1_, 33:L118δ_2_, 34:M120ε, 35:L121δ_1_, 36:L121δ_2_, 37:A129β, 38:A130β, 39:V131γ_1_, 40:V131γ_2_, 41:L133δ_1_, 42:L133δ_2_, 43:A134β, 44:T142γ_2_, 45:A146β, 46:V149γ_1_, 47:V149γ_2_, 48:I150γ_2_, 49:I150δ_1_, 50:T151γ_2_, 51:T152γ_2_, 52:T155γ_2_, 53:T157γ_2_, and 54:A160β.

The unpaired electrons of the paramagnetic triplet O_2_ induce relaxation enhancements on longitudinal and transverse spin relaxation rates. We investigated O_2_-induced longitudinal relaxation enhancements for amide and methyl protons. At 500 MHz we obtained ^1^H longitudinal relaxation rate constants, *R*_1_, for amide protons at 0 mM (Ar, 2 bar) and 6.4 mM (O_2_, 5 bar) dissolved O_2_ (see Supplementary Fig. S4 online). Figure 4a shows the paramagnetic relaxation enhancement (PRE) on ^1^H longitudinal relaxation, Δ*R*_1_, for amide protons as a function of residue number, defined as the difference of *R*_1_ between 0 mM (Ar, 2 bar) and 6.4 mM of O_2_. As an example, the relaxation curves for D89 are shown in the inset of Supplementary Fig. S4 online. As illustrated in Fig. 5a, residues exhibiting marked PREs (Δ*R*_1_ ≥ 2 s^−1^) by O_2_ are selectively observed around the two hydrophobic cavities. In contrast, PREs are small for the rest of the amide protons (Δ*R*_1_ = ~1 s^−1^). We also obtained *R*_1_ for methyl protons at different concentration of dissolved O_2_, at 600 MHz. *R*_1_ values at 0 mM (N_2_, 3 bar), 3.8 mM of dissolved O_2_ (O_2_, 3 bar), and their difference, Δ*R*_1_, are listed in Supplementary Table S1 online. Figure 6 shows O_2_-induced Δ*R*_1_ for methyl protons of the C-terminal domain (i.e., residues 71–160). Methyl protons exhibiting marked PREs (Δ*R*_1_ ≥ 4 s^−1^) were also located around the two hydrophobic cavities, as shown in Fig. 5b. Note that *R*_1_ values of several methyl protons around the two cavities could not be obtained at 3.8 mM of O_2_ concentration, because their cross-peaks were severely broadened or disappeared (see section: *The paramagnetic effect leads to line broadening*). These PRE data closely match the O_2_-induced changes in chemical shifts and peak intensities (Fig. 1).

The PRE arises from dipolar interactions between a nucleus and unpaired electrons of the paramagnet and spin relaxation contributions show < *r*^−6^ > distance dependence between the paramagnetic center and the nucleus of interest undergoing rotational motion, as described by the Solomon-Bloembergen equation^35^,^36^. O_2_-induced Δ*R*_1_ for amide protons predicted by 1/*r*^6^-weighted distance analysis is shown in Fig. 4a^13^. The crystal structure of L99A at 8 atm of xenon pressure (PDB ID, 1c6k) has three xenon atoms in cavity 4 but none in cavity 3^10^. Therefore, we added two xenon molecules (the maximum number of Xe that can be accommodated) to cavity 3 and minimized the total energy. Δ*R*_1_ was estimated from 1/*r*^6^ distance dependent PRE contribution from each xenon site, using equation (2):


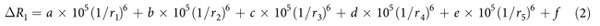


where *r*_*1–3*_ are the distances to xenon binding sites 1–3, respectively, in cavity 4 and *r*_4–5_ are the distances (Å) to the xenon binding sites 4 and 5, respectively, in cavity 3, and *a, b, c, d, e,* and *f* are fitting parameters. Hydrogen atoms were added to the crystal structure by using the WHATIF server^37^. A linear combination of predicted PREs from the five xenon-binding sites matches the observed Δ*R*_1_ pattern well (Fig. 4a). The parameters *a, b, c, d, e,* and *f* obtained from the fit were 1.3, 1.1, 1.5, 0.11, 0.10 (Å^6^/s), and 1.1 (s^−1^), respectively. Standard error of the estimate (i.e. the square root of the average squared error of prediction) was 0.74 (s^−1^). The ratio of the parameters *a, b, c, d, e,* shows the relative O_2_ occupancy at each xenon site. O_2_ occupancy in cavity 3 is about 5% of that in cavity 4. In addition, extremely large Δ*R*_1_ values were predicted for the amide protons of Y88 and L118, which indeed show severe line broadening with increasing O_2_ concentration. The *R*^2^-value of the correlation between observed and predicted Δ*R*_1_ was 0.82.

Although O_2_ occupancies at each xenon site are estimated above, contributions of each binding site to Δ*R*_1_ of each amide proton are expected to be different, as borne out by Fig. 4b. For instance, the amide protons of residues 129, 130, 153, and 154 exhibit large PREs from site 5 due to their proximity to bound oxygen molecules, even though the O_2_ occupancy at site 5 is much smaller than that for sites 1–3. The contributions to Δ*R*_1_ from the individual binding sites as a function of distance are given in Supplementary Fig. S5 online. The data can be adequately modeled with a 1/*r*^6^ distance dependence.

O_2_-induced Δ*R*_1_ for methyl protons of residues 71–160 was also predicted by the 1/*r*^6^-weighted distance analysis in Fig. 6. The *R*^2^-value of the correlation between observed and predicted Δ*R*_1_ was 0.80. These statistically substantial correlations indicate that O_2_ molecules associate with cavities 3 and 4. The parameters *a, b, c, d, e* and *f* obtained were 1.8, 0.0, 3.1, 0.0, 0.046 (Å^6^/s), and 0.79 (s^−1^) respectively. Standard error of the estimate was 3.0 (s^−1^). The predicted O_2_ occupancy in cavity 3 is much smaller than that of cavity 4, suggesting that O_2_ binding to cavity 3 is weaker than that to cavity 4. Furthermore, O_2_ occupancy at site 2 appears to be lower than that at sites 1 and 3. Although these tendencies are consistent with the results of amide protons, the ratios of parameters *a*–*e* between amide and methyl protons are different. We considered two explanations. First, the number of the data of methyl protons we used in the fit is smaller than that for amide protons. Even at a few bars of O_2_ pressure, line broadening by PREs is sufficiently large to prevent a correct estimate of Δ*R*_1_ (see section *The paramagnetic effect leads to line broadening*). Indeed, the standard error of estimate in the case of methyl protons is four times larger than that of amide protons. Alternatively, the values of the predicted Δ*R*_1_ increase very steeply, as the distance is less than 3 Å to the xenon site. For instance, even within the same methyl group, the values for each proton are largely different. Thus, in order to obtain a more quantitative prediction, we need to know the exact location and probability of O_2_ molecules at these binding sites much more precisely to calculate a correct estimate.

According to a calculation by Teng and Bryant, the O_2_-induced proton relaxation rate constant for a proton in van der Waals contact with O_2_ is about 6200 s^−1^ if O_2_ is bound at all times^12^. Accordingly, an observed value of 10 s^−1^ for Δ*R*_1_ suggests that the binding probability is ~0.2% or less. However, the O_2_-bound state probability of the protein seems to be about 5–10 times greater than the predicted binding probability according to our estimation of *K*_d_ (i.e., 1.3% at atmospheric pressure). This difference indicates that the effective distance between the O_2_ and the protein proton is larger than the van der Waals contact. We speculate that O_2_ does not tightly contact the protein proton and is allowed to move in the cavity space. We discuss the effective distance and dynamics of O_2_ in hydrophobic cavities in the section: *Rotational and translational diffusion of O*_*2*_ *in hydrophobic cavities.* Small, but substantial, Δ*R*_1_ values were observed at the residues in the N-terminal domain (i.e., residues 1–70), corresponding to the parameter *f*, and originates from O_2_ diffusing around the protein surface and proton spin diffusion, as discussed previously^38^.

### The paramagnetic effect leads to line broadening

We investigated the line-widths (Δ*ν*_1/2_) of resonance lines in the ^15^N and ^1^H dimensions of the refocused-HSQC as a function of O_2_ concentration. Because the refocused-HSQC^39^ provides line widths that are directly proportional to the transverse relaxation rate of in-phase nitrogen coherences, *R*_2_^N^, without contributions from ^1^H relaxation, it is suitable to estimate the contributions of conformational exchange and PRE contributions to the ^15^N line-width^31^. Figure S6 shows ^1^H and ^15^N line-widths for residues 85, 88, 89, 99, 100, and 118 as a function of dissolved oxygen concentration. To within experimental error, the ^15^N line widths with increasing O_2_ concentration are constant for residues around the hydrophobic cavities, while a strong increase in ^1^H line widths for the same residues is observed. This observation supports the notion that dipolar PRE is the primary cause of line broadening, as this effect is proportional to the square of the gyromagnetic ratio of the nucleus involved. The observation that all of the amide and methyl protons showing severe line broadening and loss of signal intensities are located less than 6 Å from the closest xenon binding site (Table S1) further supports this. These results show that the PRE to the transverse relaxation rates lead to the line broadening.

### Origin of O_2_-induced chemical shift change

We showed that changes in ^15^N, ^13^C, and ^1^H chemical shifts were specific to O_2_ and not observed for diamagnetic gases N_2_ and Ar. We next sought to understand whether O_2_-induced changes result mainly from the paramagnetic property of O_2_. In general, localized unpaired electrons of a paramagnet couple to the surrounding nuclei (i.e., hyperfine coupling) and may induce chemical shift changes through spin polarization and delocalization conveyed through the molecular orbitals of the molecule (contact shifts) or through the magnetic field emanating directly from the paramagnetic center (pseudocontact shifts, PCSs). In the case of O_2_, PCSs result from the anisotropic *g*-tensor of the unpaired electrons. PCSs depend on the distance between the paramagnet center and the nucleus of interest and the orientation with respect to the principal axes of the magnetic susceptibility tensor (i.e., *χ*-tensor). If PCSs were significant, the additional magnetic field would be sensed to a similar degree by the nuclei in ^15^N-^1^H and ^13^C-^1^H bonds and therefore lead to diagonal displacements of signals in ^1^H/^15^N and ^1^H/^13^C HSQC spectra, where the magnitude of change would be about the same for the bonded nuclei when measured in ppm. Such peak movement was not observed with increasing O_2_ concentration. To the contrary, ^15^N and ^13^C chemical shift changes upon O_2_ binding were much larger than ^1^H shift changes. In addition, if oxygen exchanges rapidly with the protein interior without preferred bound orientation or rapidly reorients itself with respect to the protein in the bound state, PCSs would average to zero. Instead, the O_2_ molecules in the hydrophobic cavities may have frequent collisions with nuclei. The collisions of O_2_ might allow delocalization of unpaired electron spins to ^13^C and ^15^N atoms^40^,^41^, causing contact shift. In addition, Bezsonova *et al*. showed a positive correlation between O_2_-induced chemical shift changes and increases in a collisionally accessible surface area^18^. These facts indicate that the collisions of O_2_ in the cavities could be a reasonable reason for relatively large chemical shift perturbation only for ^13^C and ^15^N.

Do N_2_ and Ar interact with the cavities of the protein? Similarities of the properties of the gases, such as mole fraction solubility, van der Waals radius and polarizability^42^,^43^, imply that N_2_ and Ar could associate to the hydrophobic cavities of L99A (Further discussions are in Supplementary Information). Indeed, binding of Ar at cavity 4 was observed in crystal structures of L99A at 8 to 32 bar of Ar pressure^10^. These results indicate that chemical shift changes by binding of noble gases, which can be attributed to changes in structure and conformational equilibrium, are in general much smaller than those by paramagnetic shifts of O_2_. Based on these observations and considerations, we conclude that O_2_-induced changes in chemical shifts resulted primarily from changes in contact shifts rather than due to the PCSs of O_2_ (see following section) and structural changes and conformational equilibria of the protein.

### Rotational and translational diffusion of O_2_ in hydrophobic cavities

**Figure 7:**
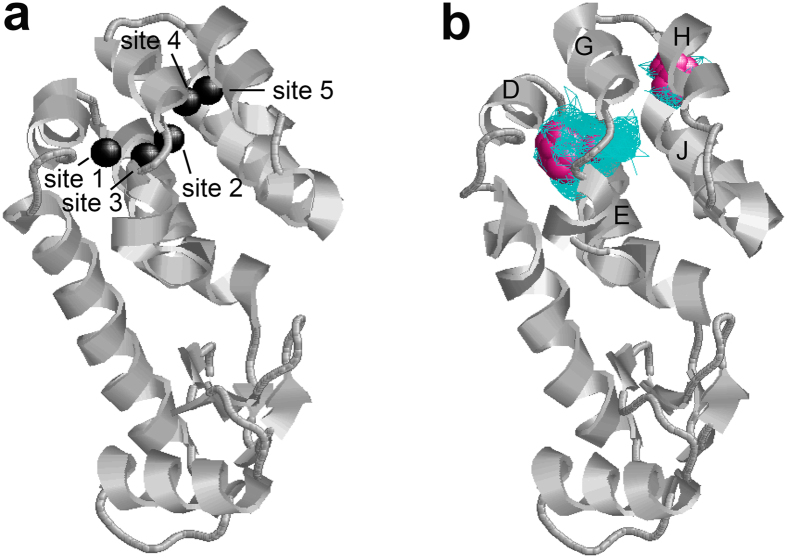
(**a**) Xenon binding sites in T4 lysozyme L99A (PDB ID; 1c6k). Cavity 4 includes xenon binding sites 1–3. We artificially created sites 4 and 5 in cavity 3 to calculate the distance to the cavity. (**b**) O_2_ density map calculated by MD simulation of 100 ns. Positions sampled by O_2_ are depicted by blue wireframe. Positions showing more than 4 times higher probabilities than the average are depicted by purple spheres. Helices D, E, G, H, and J are labeled. The picture was prepared using RasWin Molecular Graphics 2.7.5^63^.

In order to investigate rotational and translational diffusion of O_2_ in cavities 3 and 4, molecular dynamics (MD) simulations of 100 nanoseconds were performed five times. An O_2_ molecule was inserted in both cavities 3 and 4 of the crystal structure of L99A (PDB ID; 1c6k) from which the pre-existing three xenon molecules were removed. One of the MD simulations is shown in Supplementary Movie S1 online. Note that the movie consists of 500 snapshots taken every 0.2 nanoseconds. O_2_ frequently moves around each hydrophobic cavity and rotates many times within 100 ns. Similar results were obtained in the four other MD simulations. Figure 7a shows xenon binding sites 1–5 in L99A, and Fig. 7b shows a density map of O_2_ molecules in cavities 3 and 4, obtained by the 100 nanoseconds MD simulation. While O_2_ samples almost all spaces in cavity 4, sampling frequencies that were more than 4 times higher than the average were observed only at sites 1 and 3 (Fig. 7b). These results suggest that O_2_ density is substantially higher at xenon binding sites 1 and 3 than at site 2, which is qualitatively consistent with the prediction by Δ*R*_1_ for amide and methyl protons. In the small hydrophobic cavity 3, O_2_ seems to mostly populate site 5. These results are consistent with the previous one nanosecond MD simulation by Mann and Hermans^44^.

**Figure 8:**
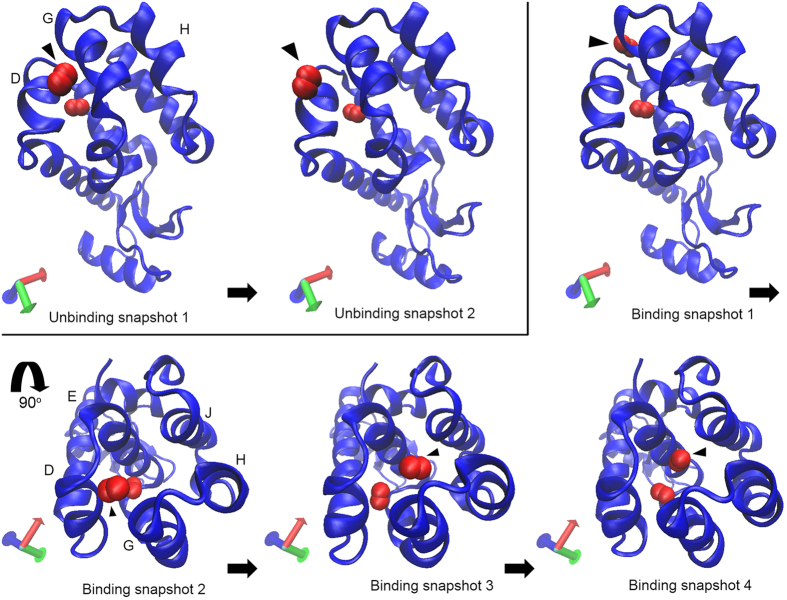
A series of snapshots showing unbinding and binding of O_2_ molecule to T4 lysozyme L99A. O_2_ molecules showing unbinding and binding to cavity 4 are indicated by filled triangles. The D, E, G, H, and J helices are labeled. The picture was prepared using VMD 1.9.2^64^.

Interestingly, in the case of one of the MD simulations (Supplementary Movie S2 online), the O_2_ molecule in cavity 3 moved into cavity 4. Such a displacement of O_2_ from cavity 3 to 4 and *vice versa* was also observed in 4 of 5 MD trajectories. Then one of the two O_2_ molecules in cavity 4 egressed from the protein through the cleft between helices D and G. Furthermore, the O_2_ molecule eventually returned to cavity 4. A series of snapshots of unbinding and binding of O_2_ are shown in Fig. 8. During the O_2_ binding process, the O_2_ molecule binds to the surface near the helices D and G first and then returns to cavity 4 through the center of the channel consisting of helices D, E, G, H, and J. In the other simulation (Supplementary Movie S3 online), we also observed unbinding of O_2_ from cavity 3 through the cleft between helices H and J, as seen in Supplementary Fig. S7 online. Although it is known that L99A allows xenon and benzene to bind to cavity 4, the pathway of ligand access and egress is unknown. The present results provide the first insights in the potential pathway of ligand binding and unbinding to cavity 4, as well as egress from cavity 3.

We separately performed additional one nanosecond MD simulations to understand more details of the rotational diffusion of O_2_ in cavity 4. A time dependent relaxation of the rotational correlation function was more reasonably fitted to a bi-exponential function than a single-exponential one, as shown in Supplementary Fig. S8 online. Rotational correlation times estimated by a bi-exponential function were 0.164 ± 0.006 ps and 1.41 ± 0.02 ps. Such a bimodal rotational correlation is well known for small molecules in many solvents^45^,^46^. The fast and slow components would be related to the inertial characteristics of the small molecule and diffusive solvent motions, respectively^45^,^46^. Therefore, we speculate that the fast and slow components in the present case are related to the inertial characteristic of O_2_ and protein internal dynamics taking place around the cavity, respectively. A different explanation is anisotropy of rotational motion of O_2_ in the cavity. In any case, it appears that the rotational motions taking place at sub-nanosecond will average the orientation with respect to the principal axes of the magnetic susceptibility tensor and reduce the PCSs. On the other hand, chemical shifts can be changed with an increase in a population of “the O_2_-bound state”. Because the observed NMR signals are the ensemble average of exchanging conformations, O_2_-induced Δ*R*_1_ of a particular proton would depend on the averaged-distance from the O_2_ molecule, which would be larger than the van der Waals contact. These results strongly support our discussions on PREs and PCSs.

### O_2_ associates selectively and preferentially to hydrophobic and not to hydrophilic internal protein cavities

Although the size of hydrophilic cavities (cavities 1 and 2) is considered to be enough for O_2_ binding, we could not detect O_2_-induced changes in spectral parameters, such as chemical shifts, peak line-widths, and longitudinal relaxation rate constants, for residues around these cavities. Interestingly, X-ray crystallography found electron density in the hydrophobic and hydrophilic cavities (cavities 1, 2, and 4), which are attributed to water molecules. In hydrophilic cavity 1, two well-ordered water molecules were seen, while a single well-ordered water molecule was seen in hydrophilic cavity 2. In hydrophobic cavity 4, weak electron density was distributed around the cavity^47^. In contrast, no gas molecules or water molecules have previously been detected in cavity 3, as far as we are aware. O_2_ binding to cavities is generally considered to be in competition with water binding. Water molecules associate to the hydrophilic cavities more than O_2_ probably due to its dipolar property and higher concentration (i.e., ~55.6 M) in solution. Therefore, the observation of O_2_ penetration into the hydrophilic cavities of proteins is expected to be difficult. The present data show that O_2_ penetrates into the protein interior and selectively and preferentially associates with the two hydrophobic cavities of the protein. The preferential partitioning of O_2_ into hydrophobic regions is consistent with earlier observation of O_2_ binding to ribonuclease A^12^ and lipid bilayer^18^, for example.

### O_2_ association with the hydrophobic cavities and dynamic motion of the protein

Penetration of O_2_ into the protein interior requires spaces greater than its molecular size, such as a cavity, and transient conformational fluctuations, which provide pathways for penetration. Nucleus-electron dipolar interactions may therefore be modulated by conformational fluctuations of the protein and depend on the solubility of O_2_ in the protein interior. Our previous work by high-pressure NMR spectroscopy revealed that T4 lysozyme L99A has at least two types of conformational fluctuations^6^; one takes place within the ground state ensemble, which is limited to the C-terminal domain. These fluctuations occur more rapidly than a millisecond and provide heterogeneous conformations around the hydrophobic cavities. The conformational fluctuations within the ground state ensemble may allow a penetration of gas molecules into the protein interior. Indeed, MD simulations showed that O_2_ molecules frequently move around cavities 3 and 4 and may go back and forth between the cavities and the outside of the protein during 100 ns. A second motion present for T4 lysozyme L99A is a conformational fluctuation between the ground state and a transiently formed high-energy “excited” state of the protein, which takes place on the millisecond time scale (average 0.7 ms)^30^. Because the aromatic side chain of F114 flips into the enlarged cavity in the excited state, O_2_ association will compete with the F114 flip-in in this excited state. Finally, O_2_-induced changes in chemical shifts and signal intensities were also observed for some methyl groups around cavity 4 (39 Å^3^) of cysteine-free wild-type (C54T/C97A; WT*) T4 lysozyme (see Supplementary Fig. S9 online). These observations indicate that O_2_ molecules associated to the cavity 4 of WT* in the absence of a conformational fluctuation between ground and transiently formed excited states. Taken together, these data suggest that O_2_ association is facilitated by the conformational fluctuation taking place within the ground state ensemble, rather than between the ground and excited states of L99A, and that the transiently formed excited state is not involved in gas binding to the enlarged cavity. The fact that quenching of the tryptophan fluorescence by O_2_ occurs on nanosecond time scale for several native proteins^16^,^17^ agrees with this conclusion.

## Conclusion

Gas-pressure NMR spectroscopy using O_2_ has been used to explore dynamic cavities in T4 lysozyme L99A. We have come to the following conclusions:

1. O_2_ preferentially interacts with hydrophobic cavities and induces significant changes in NMR spectra, such as increased peak-widths and longitudinal relaxation rate constants, due to its paramagnetic property.
2. O_2_ associates to the two hydrophobic cavities in T4 lysozyme L99A. So far, no gas or water molecules have been detected in cavity 3. The present study provides the first evidence of ligand binding to cavity 3.
3. O_2_-induced relaxation enhancements could be adequately accounted for by the paramagnetic dipolar relaxation, assuming 1/*r*^6^-weighted contributions from five sites, where *r* is the distance to the paramagnet.
4. The dissociation constant for O_2_ binding to cavity 4 of the protein is 21 mM, indicating that about 1% of the protein contains O_2_ molecules in the dynamic hydrophobic cavity at ambient pressure.
5. According to MD simulations, O_2_ molecules in the hydrophobic cavities of the protein frequently move and rotate on the picosecond to nanosecond time scale. The cleft between helices D and G and the channel consisting of helices D, E, G, H, and J could be the potential gateway for ligand binding to cavity 4. O_2_ association with the hydrophobic cavities would be facilitated by the conformational fluctuations taking place within the ground state ensemble, rather than between the conformational ground and excited states of the protein.
6. The rotational and translational motions of O_2_ in the hydrophobic cavities may effectively reduce potential pseudocontact shift contributions to nuclear shielding.

The combination of NMR and MD simulation provides static and dynamic aspects of O_2_ binding to hydrophobic cavities. This approach might also be useful to probe the permeation pathway of ions or small molecules, such as channel-blocking molecules in membrane proteins^48^ and hydrophobic binding pockets for ligands, including drug compounds. Knowledge of protein permeation by oxygen is also highly relevant for optical spectroscopy and microscopy, where O_2_ dissolved in the protein matrix leads to quenching and bleaching, and knowledge of oxygen association pockets may facilitate the elimination of oxygen-free cavities through protein engineering. This strategy has the potential to greatly improve our understanding of the role played by protein cavities in biologically relevant processes.

## Methods

### Sample preparation

T4 lysozyme L99A was prepared from the recombinant cysteine-free T4 lysozyme (WT*, C54T/C97A)^31^. Uniformly ^15^N-labeled or ^15^N/^13^C-labeled L99A was produced in M9 media with ^15^NH_4_Cl and ^13^C_6_ glucose as the sole nitrogen and carbon sources, following established protocols^31^. The purified protein sample was dialyzed in 50 mM phosphate buffer including 25 mM NaCl at pH 5.5. Sample concentration was measured by UV absorption at 280 nm and was calculated with a molar extinction coefficient of 25440 M^−1^cm^−1^ at 280 nm.

### NMR experiments

We used ^1^H 500 MHz (Bruker BioSpin Co. AVANCE ΙΙΙ) or 600 MHz (Bruker BioSpin Co. AVANCE) NMR spectrometers. In order to study the binding of oxygen (O_2_), nitrogen (N_2_), and argon (Ar) to the protein, we used a pressure resistance NMR tube (528-QPV-7, Wilmad-Lab Glass Co.) connected to a gas cylinder by PTFE tubing. Gas pressure was applied to adjust their concentrations in the protein solution. Mole fraction solubility of O_2_, N_2_, and Ar in water are 2.3 × 10^−5^, 1.2 × 10^−5^, and 2.5 × 10^−5^, respectively, at 298 K^43^. In this article, we use absolute pressure (gauge pressure + atmospheric pressure). ^1^H-NMR, ^1^H/^15^N refocused-HSQC^39^, and ^1^H/^13^C CT-HSQC spectra were obtained for 0.50 mM uniformly ^15^N-labeled or 1.0 mM ^15^N/^13^C-labeled T4 lysozyme L99A solution at 298 K at different gas pressures. ^1^H longitudinal relaxation enhancements for amide and methyl protons were obtained from ^1^H/^15^N HSQC and ^1^H/^13^C CT HSQC spectra using saturation-recovery, achieved with proton x and y purge pulses followed by a relaxation delay before each scan^49^. Seven to ten relaxation delays ranging from 0.003 s to 1.5 s were used. Spectral analysis was performed using NMRPipe^50^ and Sparky^51^.

### Molecular dynamics simulation

Molecular dynamics (MD) simulations of 100 nanoseconds were performed five times using GROMACS 4.6.4 simulator^52^. The system contained a T4 lysozyme L99A (PDB ID; 1c6k), two O_2_ molecules, 8 chloride ions, and about 15,000 water molecules. Three xenon molecules in cavity 4 of the protein were removed from the structure, and one O_2_ molecule was inserted in each hydrophobic cavity (i.e., cavities 3 and 4). The OPLSLL force field^53^ was used for the protein, and the TIP4P model was used for water^54^. Potential parameters for O_2_ and chloride ions were as described in the literature^55^,^56^. MD simulations were conducted with the *NPT* ensemble (300 K, 1 bar) in a truncated dodecahedron box with dimensions of 25.8 Å. Temperature was controlled using a Langevin thermostat with a viscosity of 0.5 ps^−1^. Pressure was controlled by a Parrinello−Rahman barostat with relaxation times of 2.0 ps^57^. Electrostatics were treated using the particle mesh Ewald (PME) method with a 10.0 Å cutoff distance^58^. The van der Waals interactions were expressed using the twin-range cutoff method with 10.0 and 12.0 Å distances. Covalent bonds for hydrogen atoms in the polypeptide were constrained using the linear constraint solver (LINCS)^59^. Covalent bonds in water were constrained using the SETTLE algorithm^60^. The integration time step was 2 femtoseconds.

In order to estimate the rotational correlation times of O_2_ in cavity 4, we performed separate one nanosecond MD simulations. To avoid artifacts from the thermostat and barostat, we carried out the MD simulations in the *NVE* ensemble with the structure after 100 ns simulation in the *NPT ensemble*^61^. Snapshots were recorded every 0.01 picoseconds. Rotational correlation times were calculated using 1 ns trajectories of the O_2_ molecule in cavity 4. We defined a direction vector between the two oxygen atoms relative to the orientation of the protein.

## Additional Information

**How to cite this article**: Kitahara, R. *et al*. Detecting O_2_ binding sites in protein cavities. *Sci. Rep.* **6**, 20534; doi: 10.1038/srep20534 (2016).

## Supplementary Material

Supplementary Information

Supplementary Movie S1

Supplementary Movie S2

Supplementary Movie S3

## Figures and Tables

**Table 1:** Chemical shift changes of representative amide nitrogen, methyl protons, and methyl carbons for the oxygen binding to L99A.

nucleus	Δδ_max/ppm^a^	Std. Error
K85 N	0.8	0.12
Y88 N	5.3	0.7
D89 N	0.5	0.10
A99 N	2.1	0.2
I100 N	0.6	0.10
L118 N	0.8	0.12
I78 Hδ_1_	0.39	0.06
I78 Hγ_2_	0.32	0.06
M102 Hε	0.61	0.08
I78 Cδ_1_	2.3	0.2
I78 Cγ_2_	2.7	0.3
M102 Cε	3.9	0.4

^a^Δ*δ* are obtained by a global fitting for changes in chemical shifts using eq. 1.
