# Supplementary material for: Detecting O2 binding sites in protein cavities
Source: Sci Rep. 2016 Feb 2;6:20534. doi: 10.1038/srep20534 (PMC4735865; doi:10.1038/srep20534)
Supplement: Supplementary Information [file srep20534-s1.pdf]

Supplementary Information

Detecting O_2 binding sites in protein cavities

Ryo Kitahara[1,*], Yuichi Yoshimura[2], Mengjun Xue[2], Tomoshi Kameda[3], Frans A. A. Mulder[2,*]

[1]College of Pharmaceutical Sciences, Ritsumeikan University, Nojihigashi 1-1-1, Kusatsu 525-8577, Japan, [2]Department of Chemistry and Interdisciplinary Nanoscience Center (iNANO), University of Aarhus, Gustav Wieds Vej 14, DK-8000 Aarhus C, Denmark, [3]Biotechnology Research Institute for Drug Discovery, Advanced Industrial Science and Technology, 2-43 Aomi, Koto, Tokyo 135-0064, Japan

Supplementary materials: Discussion, Figure S1-S9, Table S1, and Movie S1-S3

Discussion

Diffusion of O_2 into protein solution in the NMR tube.

Supplementary Figure S1 shows time-dependent changes in ^1H NMR spectra of ^{15}N labeled T4 lysozyme L99A when the oxygen concentration decreased from 1.8 mM to 0.27 mM over 18.7 hours at 298 K. A well-separated peak of L121 $H\delta_1$ changed its frequency (about 0.05 ppm) during 18.7 hours. We consider that this is due to the slow rate of diffusion of O_2 into protein solution in the NMR tube (inner diameter: 4 mm). Indeed, the time to reach equilibrium could be shortened when shaking or increasing surface area was employed (data not shown). Out of safety considerations, we did not shake the pressurized NMR tube in the present experiments.

The potential of O_2, N_2 and Ar gas molecules binding to protein cavities.

The mole fraction solubilities of O_2, N_2, and Ar in water at 298 K are 2.3×10^{-5}, 1.2×10^{-5}, and 2.5×10^{-5}, respectively[1]. All values refer to the partial pressure of gas at one atmosphere. The van der Waals radius of Ar is 188 pm, whereas the length of the major axis of O_2 and N_2 are 218 pm and 226 pm, respectively, indicating that all three gases would fit to similar size cavities. However, in determining whether these gases possess affinity for protein cavities, their polarizability needs to be considered. It is known, for example, that the polarizability of noble gases is correlated to their affinity to protein cavities. Because electric dipole polarizabilities of N_2 and Ar are similar to that of oxygen (N_2:1.74, Ar: 1.64, O_2: 1.57 in units of 10^{-24} cm^3)[2], they should be as likely to interact with the cavities as O_2.

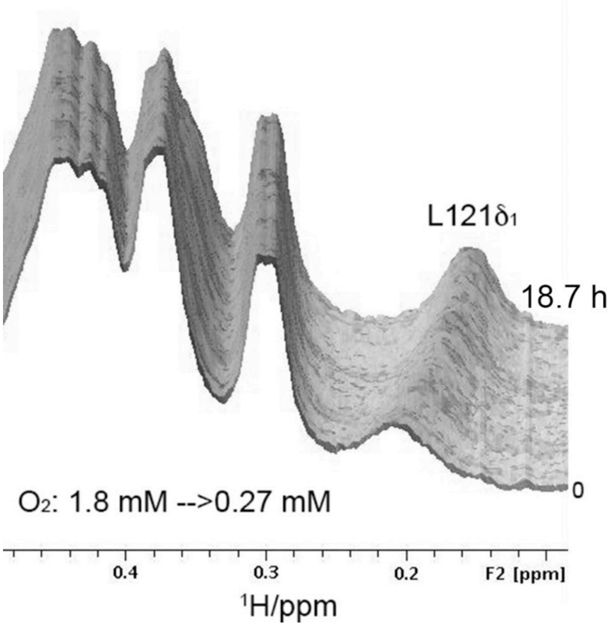

Figure S1. Time-dependent changes in ^{1}H NMR spectra of ^{15}N labeled T4 lysozyme L99A, when the oxygen concentration decreased from 1.8 mM to 0.27 mM over 18.7 hours at 298 K. The peak of L121 Hδ_1 is indicated.

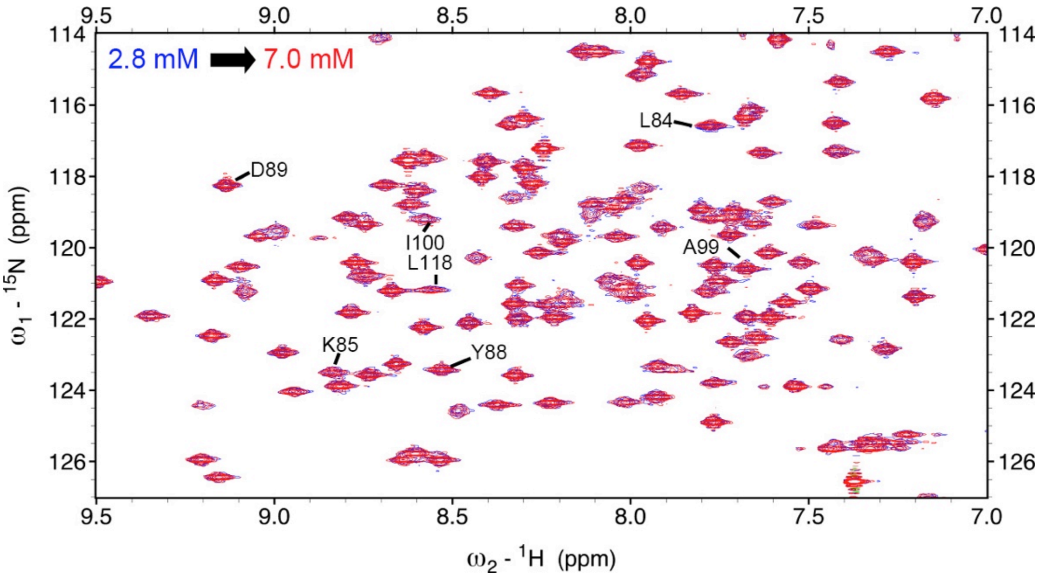

Figure S2. $^{1}H/^{15}N$ HSQC spectra obtained for ^{15}N labeled T4 lysozyme L99A at 2 bar (corresponding to 2.8 mM) and 5 bar (corresponding to 7.0 mM) Ar.

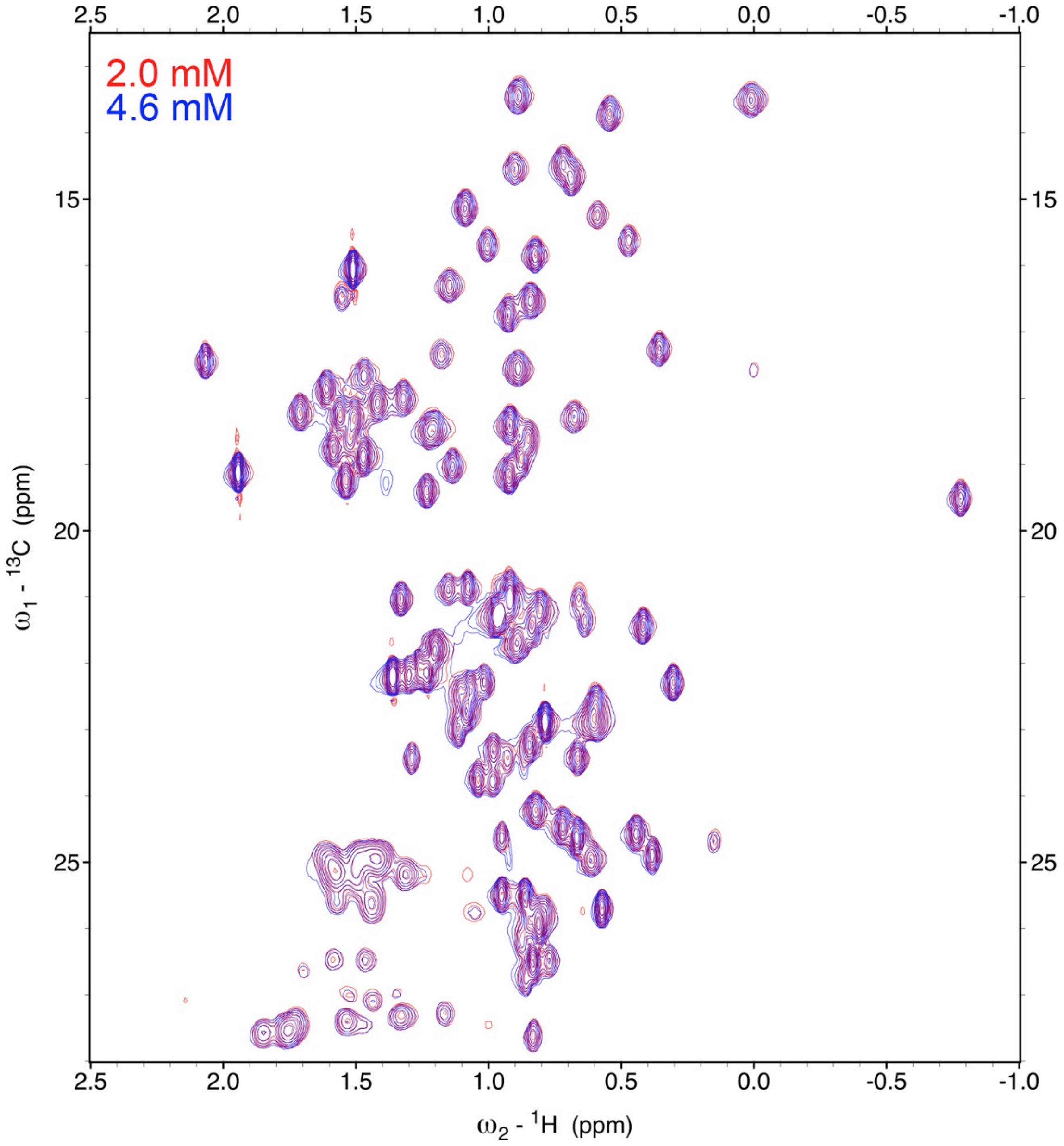

Figure S3. $^1H/^{13}C$ CT HSQC spectra obtained for $^{15}N/^{13}C$ labeled T4 lysozyme L99A at 3 bar (corresponding to 2.0 mM) and 7 bar (corresponding to 4.6 mM) N_2.

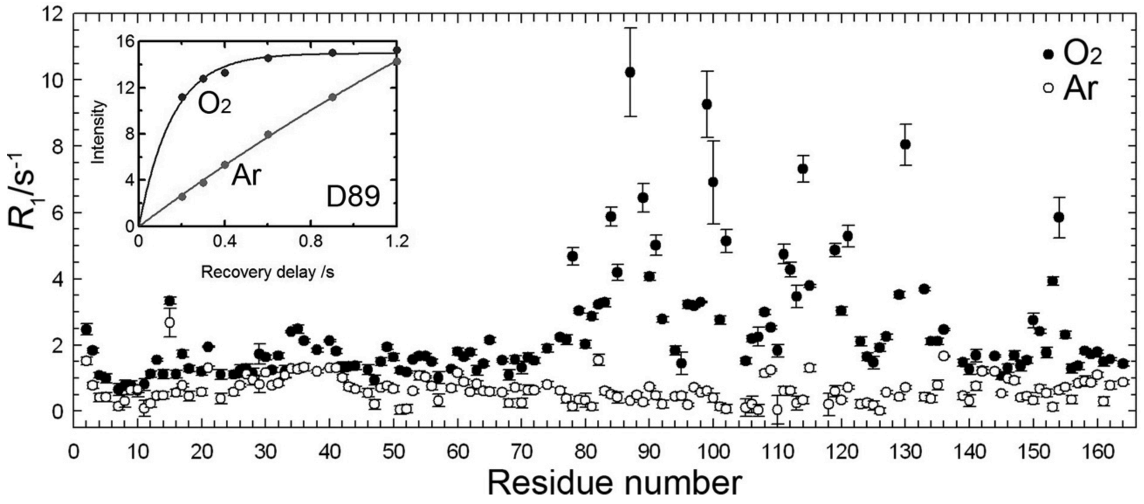

Figure S4. ^{1}H longitudinal relaxation rate constants, R_1, for amide protons at 0 mM (Ar 2 bar, open circles) and 6.4 mM (O_2 5 bar, closed circles) O_2 against residue number. Error bars from curve fitting are included. Changes in crosspeak intensities of D89 in ^{1}H/^{15}N HSQC spectra as a function of recovery delay are shown in the inset.

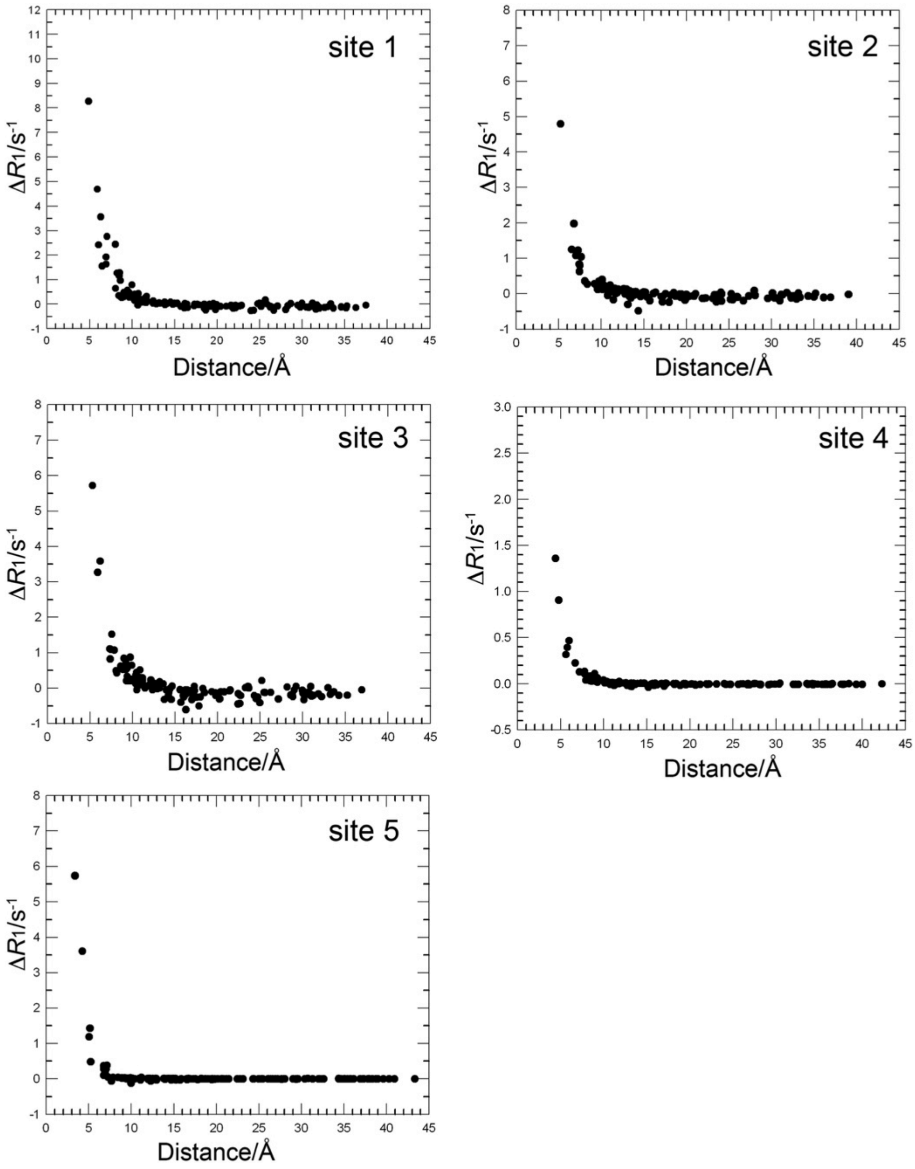

Figure S5. Predicted ΔR_1 for amide protons due to paramagnetic relaxation at each binding site (1-5) as a function of distance. ΔR_1 from each binding site 1-5 is explained in Figure 4b.

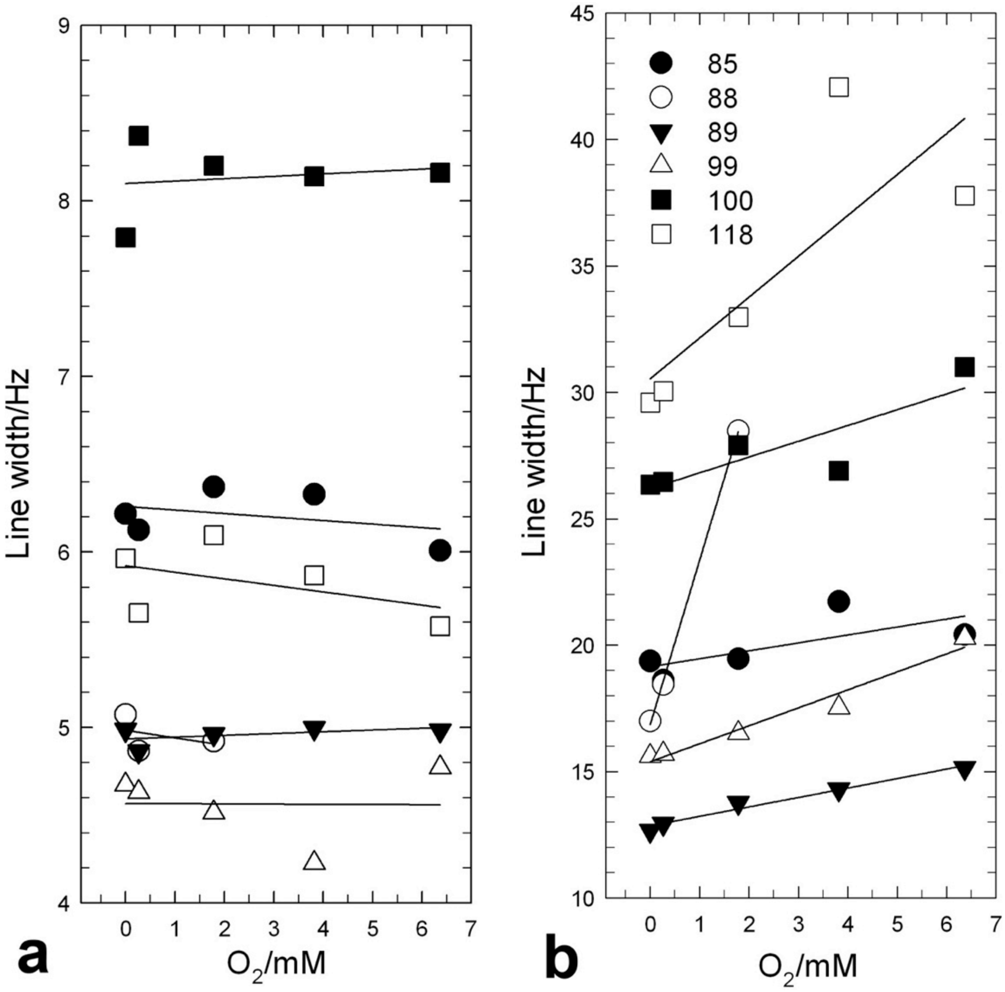

Figure S6. ^{15}N (a) and ^{1}H (b) line-widths of crosspeaks of the residues around the enlarged cavity in the refocused ^{1}H/^{15}N HSQC spectra. The legend included in panel b is also applicable to panel a.

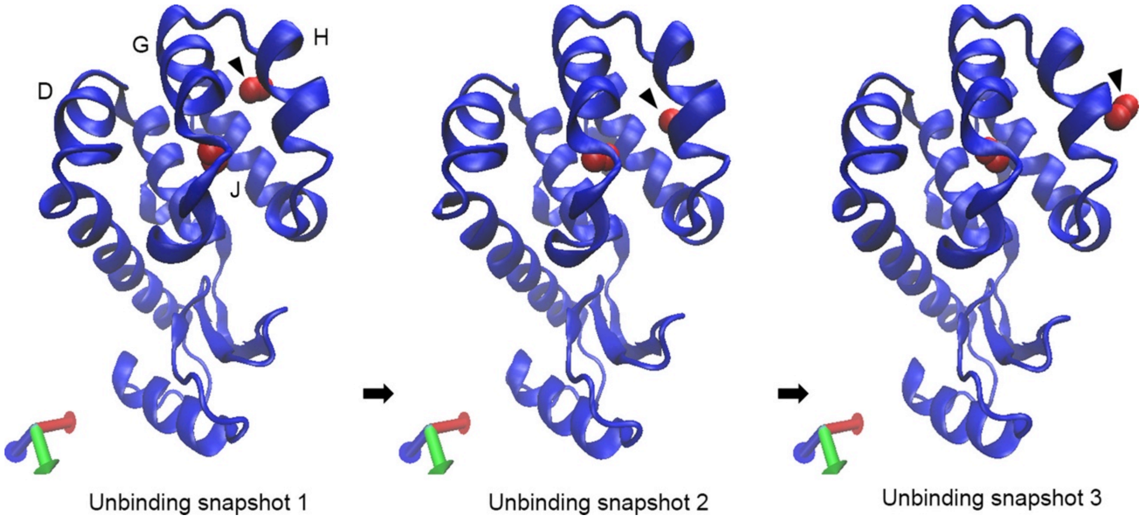

Figure S7. A series of snapshots showing egress of O_2 molecule from T4 lysozyme L99A. O_2 molecules showing egress from cavity 3 are indicated by filled triangles. The D, G, H, and J helices are labeled. The picture was prepared using VMD 1.9.2.

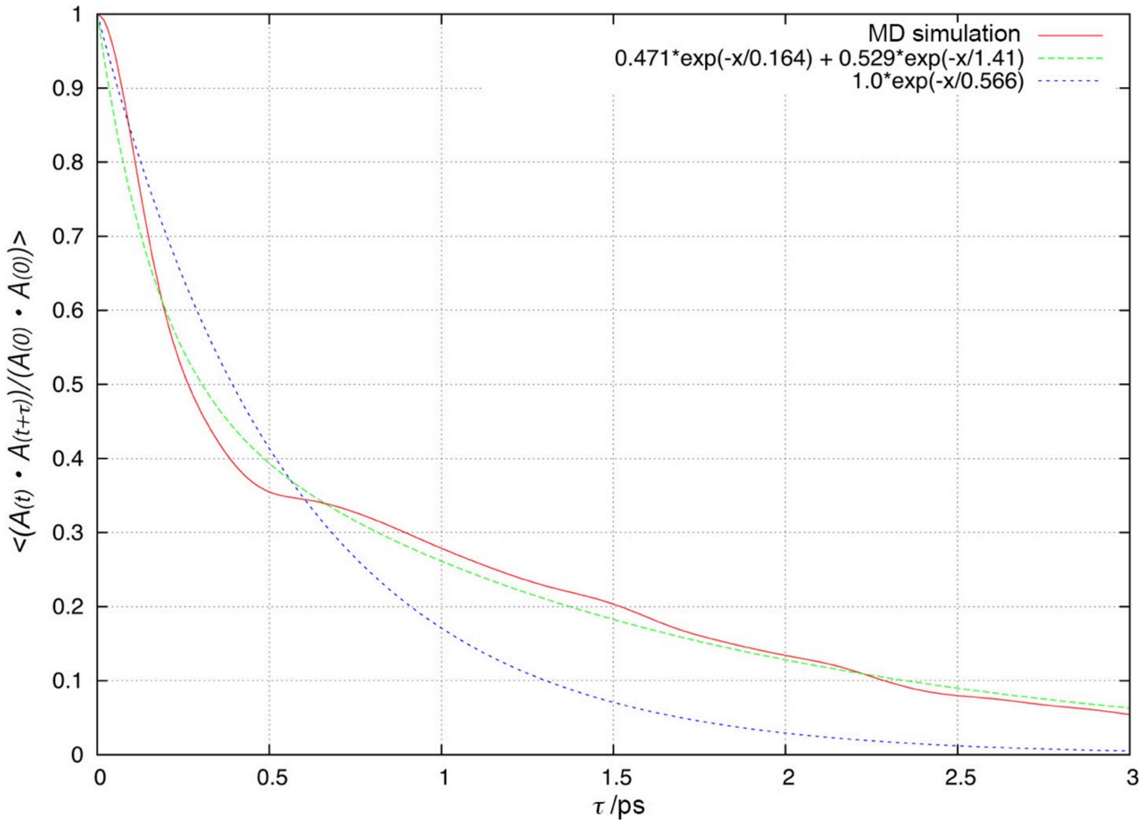

Figure S8. Estimation of the rotational correlation time for O_2 in cavity 4. $A(t)$ and $A(t+\tau)$ are direction vectors joining the two oxygen atoms at time t and $t+\tau$, respectively. $<(A(t)\bullet A(t+\tau))/(A(0)\bullet A(0))>$ shows rotational correlation function of O_2 molecule. Assuming single-exponential (blue) or bi-exponential (green) decays, the rotational correlation times of O_2 were estimated to be 0.6±0.1 ps or 0.164±0.006 ps and 1.41±0.02 ps, respectively.

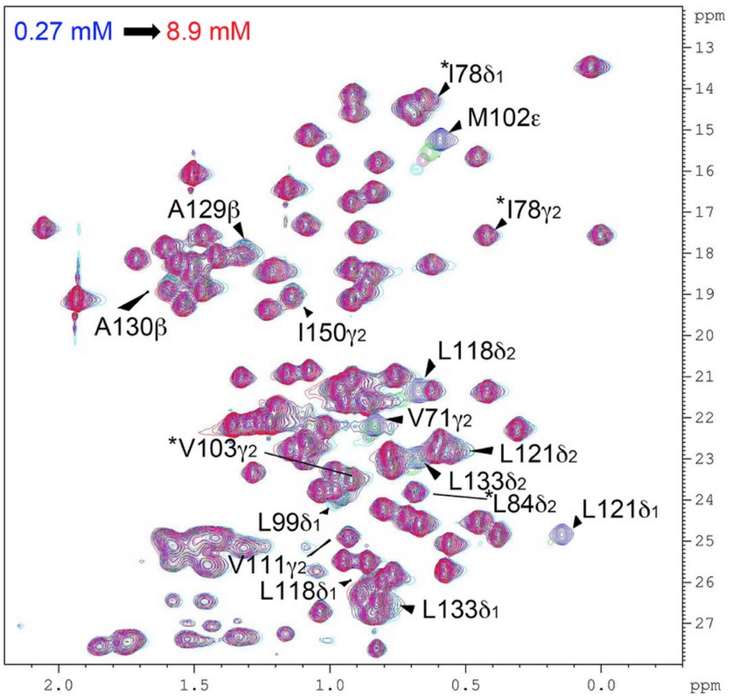

Figure S9. $^1H/^{13}C$ constant time HSQC spectra of $^{13}C/^{15}N$ labeled T4 lysozyme (WT*) at different oxygen concentrations from 0.27 mM to 8.9 mM. Positive and negative crosspeaks are presented by same color. Methyl groups showing significant changes in $^1H/^{13}C$ chemical shift and a loss of crosspeak intensities on L99A variant are indicated. Asterisks show methyl groups which exhibit significant change in chemical shifts and/or crosspeak intensities in L99A but not in WT* protein.

Table S1. O_2-induced longitudinal relaxation enhancements of methyl protons.

Group	O_2 (3 bar)[a] R_1/s^{-1}	Error	N_2 (3 bar)[a] R_1/s^{-1}	Error	ΔR_1/s^{-1}	Error	Distance/Å[c]
M1ε	1.49	0.04	0.95	0.06	0.54	0.07	19
I3γ_2	2.4	0.5	1.3	0.4	1.1	0.7	11
I3δ_1	2.5	0.16	1.5	0.13	1.0	0.2	14
M6ε	1.12	0.09	0.6	0.11	0.6	0.14	11
L7δ_1	2.6	0.3	2.4	0.3	0.2	0.5	13
L7δ_2	2.5	0.16	2.5	0.15	0.0	0.2	13
I9γ_2	2.2	0.2	1.9	0.18	0.3	0.3	19
I9δ_1	1.9	0.19	1.6	0.3	0.3	0.3	20
L13δ_1	2.0	0.11	1.6	0.12	0.4	0.17	26
L15δ_1	3.0	0.2	2.7	0.14	0.3	0.3	30
I17γ_2	2.8	0.16	2.2	0.13	0.7	0.2	30
I17δ_1	2.1	0.2	1.5	0.17	0.6	0.3	29
I27δ_1	2.0	0.19	1.9	0.10	0.2	0.2	29
I27γ_2	2.7	0.3	2.5	0.19	0.2	0.3	25
I29γ_2	1.6	0.19	1.2	0.3	0.4	0.3	19
I29δ_1	1.7	0.19	1.4	0.2	0.3	0.3	22
L32δ_1	2.1	0.10	1.7	0.08	0.5	0.13	21
L32δ_2	2.6	0.10	2.3	0.14	0.3	0.17	19
L33δ_2	3.8	0.4	2.6	0.3	1.2	0.5	26
L33δ_1	3.4	0.4	3.6	0.6	-0.2	0.7	25
T34γ_2	3.0	0.2	3.0	0.3	0.0	0.4	27
L39δ_1	2.7	0.2	2.4	0.2	0.3	0.3	35
L39δ_2	3.5	0.2	2.3	0.13	1.2	0.3	31
A41β	4.1	0.3	4.1	0.3	0.0	0.5	32
A42β	2.3	0.18	2.1	0.2	0.2	0.3	29
L46δ_1	3.0	0.3	2.4	0.3	0.6	0.4	28
L46δ_2	2.9	0.5	2.6	0.4	0.3	0.6	30
A49β	2.7	0.16	2.2	0.17	0.5	0.2	26

I50γ_2	2.6	0.17	2.0	0.15	0.6	0.2	31
I50δ_1	2.6	0.16	1.4	0.17	1.2	0.2	28
T54γ_2	2.3	0.15	1.9	0.10	0.4	0.18	33
V57$\gamma_{1\&2}$	2.93	0.06	2.33	0.05	0.60	0.08	35
I58γ_2	1.9	0.16	1.7	0.16	0.2	0.2	30
I58δ_1	2.0	0.2	1.8	0.3	0.2	0.3	27
T59γ_2	3.0	0.11	2.4	0.14	0.6	0.18	34
A63β	2.6	0.3	2.3	0.3	0.3	0.4	25
L66δ_1	3.0	0.3	2.0	0.2	1.0	0.4	24
L66δ_2	3.3	0.2	1.9	0.16	1.4	0.3	24
V71γ_2	2.9	0.2	2.4	0.16	0.6	0.3	15
A73β	4.4	0.3	2.0	0.12	2.4	0.4	16
A74β	4.2	0.6	2.4	0.5	1.8	0.8	11
V75γ_1	4.6	0.3	2.6	0.18	2.0	0.3	11
V75γ_2	3.8	0.3	1.7	0.19	2.1	0.4	11
I78γ_2	32	10	1.2	0.17	31	10	4.6
I78δ_1	21	6	0.9	0.12	20	6	5.5
L79δ_2	3.7	0.3	1.8	0.14	1.9	0.3	8.4
A82β	3.2	0.16	2.2	0.12	1.0	0.2	12
L84δ_1	15	1.8	2.5	0.6	12	1.9	5.7
L84δ_2	b		2.9	0.2			4.3
V87γ_1	e		2.6	0.5			4.2
V87γ_2	8.3	0.3	1.2	0.11	7.1	0.3	5.4
L91δ_2	9.9	0.8	1.0	0.19	8.9	0.8	7.2
A93β	3.9	0.3	2.9	0.19	0.9	0.3	14
V94γ_1	1.7	0.16	1.3	0.17	0.5	0.2	11
V94γ_2	2.1	0.14	1.4	0.13	0.7	0.19	14
A97β	4.2	0.4	2.5	0.4	1.7	0.6	12
A98β	3.6	0.4	1.4	0.3	2.1	0.5	6.9
A99β	b		3.5	0.8			3.6
I100γ_2	3.4	0.3	1.7	0.3	1.7	0.4	9.9
I100δ_1	4.1	0.3	1.2	0.14	2.9	0.3	9.4

M102ε	23	7	0.7	0.3	22	7	4.4
V103γ_1	4.9	0.3	2.9	0.19	2.0	0.3	8.5
V103γ_2	e		1.8	0.5			5.4
M106ε	2.4	0.2	0.6	0.15	1.8	0.3	7.9
T109γ_2	3.4	0.19	2.6	0.13	0.8	0.2	13
V111γ_1	e		2.4	0.19			3.3
V111γ_2	10	2	2.3	0.4	8	2	5.7
A112β	4.5	0.3	2.0	0.2	2.5	0.4	8.6
T115γ_2	3.8	0.3	3.0	0.16	0.9	0.3	9.9
L118δ_1	3.6	0.16	2.4	0.3	1.3	0.3	5.6
L118δ_2	b		2.1	0.5			3.8
M120ε	1.14	0.04	0.6	0.03	0.58	0.05	8.2
L121δ_1	b		2.4	1			3.5
L121δ_2	2.8	0.18	2.0	0.12	0.9	0.2	3.7
A129β	8.8	0.7	1.6	0.2	7.2	0.8	3.5
A130β	9.3	0.9	2.3	0.2	6.9	0.9	4.1
V131γ_1	3.2	0.11	2.44	0.09	0.7	0.14	9.8
V131γ_2	1.70	0.08	1.45	0.09	0.3	0.12	8.6
L133δ_1	12	3	2.6	0.4	9	3	3.5
L133δ_2	4.7	0.8	3.2	0.3	1.4	0.9	3.7
A134β	2.6	0.2	1.8	0.2	0.8	0.3	8.8
T142γ_2	3.5	0.4	2.8	0.5	0.7	0.6	16
A146β	3	1	4	2	-1	2	10
V149γ_1	3.6	0.3	1.5	0.17	2.2	0.3	7.6
V149γ_2	2.3	0.3	1.5	0.3	0.8	0.4	9.8
I150γ_2	6.0	0.6	1.5	0.2	4.5	0.6	4.5
I150δ_1	2.6	0.4	1.2	0.3	1.4	0.4	6.8
T151γ_2	2.88	0.08	2.39	0.08	0.5	0.12	9.7
T152γ_2	2.7	0.4	2.4	0.5	0.3	0.6	9.3
T155γ_2	2.8	0.17	2.3	0.2	0.6	0.3	9.2
T157γ_2	2.8	0.2	2.3	0.17	0.5	0.3	13
A160β	2.28	0.09	2.1	0.14	0.2	0.17	11

L164δ_1	3.1	0.13	2.5	0.10	0.6	0.16	d
L164δ_2	2.70	0.07	2.20	0.07	0.5	0.10	d

[a]Absolute pressure consisting of gauge pressure plus atmospheric pressure.

[b]Crosspeaks severely broadened at 3.8 mM O_2.

[c]Distances from the closest xenon (one of sites 1-5) to each hydrogen in the L99A crystal structure at 8 atm xenon pressure (PDB ID, 1c6k). Hydrogen atoms were added to the crystal structure by using the WHATIF server.

[d]Atomic position is not available.

[e]Peaks are overlapped with other peaks.

Movie S1. MD simulation of 100 nanoseconds (example 1). An O_2 molecule was inserted in both cavities 3 and 4 of the crystal structure of L99A T4 lysozyme (PDB ID; 1c6k). The movie consists of 500 snapshots taken every 0.2 nanoseconds.

Movie S2. MD simulation of 100 nanoseconds (example 2). An O_2 molecule was inserted in both cavities 3 and 4 of the crystal structure of L99A T4 lysozyme (PDB ID; 1c6k). The movie consists of 500 snapshots taken every 0.2 nanoseconds.

Movie S3. MD simulation of 100 nanoseconds (example 3). An O_2 molecule was inserted in both cavities 3 and 4 of the crystal structure of L99A T4 lysozyme (PDB ID; 1c6k). The movie consists of 500 snapshots taken every 0.2 nanoseconds.

References

1. Miller, T.M. CRC Handbook of Chemistry and Physics, 95th Edition, Vol. 95. (CRC Press, 2014).
2. Scharlin, P., Battino, R., Silla, E., Tunon, I. & Pascual-Ahuir, J.L. Solubility of gases in water: Correlation between solubility and the number of water molecules in the first solvation shell. *Pure Appl. Chem.* **70**, 1895-1904 (1998).
